# Case report: Schnitzler-like syndrome without monoclonal gammopathy

**DOI:** 10.3389/fimmu.2023.1166620

**Published:** 2023-03-30

**Authors:** Anna Sophie Wesselmann, Axel Künstner, Anke Fähnrich, Christian Rose, Peter Lamprecht, Hauke Busch, Ralf J. Ludwig, Andreas Recke

**Affiliations:** ^1^ Department of Dermatology and Allergy, University of Lübeck, Lübeck, Germany; ^2^ Lübeck Institute of Experimental Dermatology, University of Lübeck, Lübeck, Germany; ^3^ Department of Rheumatology and Clinical Immunology, University of Lübeck, Lübeck, Germany

**Keywords:** case report, Schnitzler’s syndrome, autoinflammation, late-onset autoinflammation, gammopathy

## Abstract

Schnitzler syndrome is a rare autoinflammatory disorder characterized by urticarial rash, joint pain, recurrent fever, leucocytosis, elevated C-reactive protein (CRP) and serum amyloid A (SAA), and monoclonal IgM or IgG gammopathy. According to the *Strasbourg criteria*, both urticarial rash and gammopathy are mandatorily required for the diagnosis of Schnitzler’s syndrome. However, incomplete variants lacking either skin symptoms or monoclonal gammopathy have also been described. Here, we report a case in which the diagnosis of Schnitzler-like syndrome was made despite the absence of gammopathy, based on neutrophilic dermal inflammation, episodic and excessive increase in inflammatory parameters, and prompt response to anakinra, a soluble IL1 receptor antagonist (sIL-1RA). In addition, we detected neutrophil epitheliotropism, which is highly suggestive of autoinflammatory disease. Using whole-exome sequencing, we were unable to find a causative pathogenic mutation but did find several mutations possibly related to the inflammatory processes in this patient. This and other cases highlight that the existing *Strasbourg criteria* are too strict to capture Schnitzler-like syndromes that may respond well and rapidly to IL1 inhibition. Recurrent episodes of disease with normalization of inflammatory symptoms in the interval, rapid response to anakinra, and neutrophilic epitheliotropism in a lesional skin biopsy may help confirm the diagnosis of Schnitzler-like syndrome.

## Introduction

Schnitzler`s syndrome is a rare autoinflammatory disease characterized by urticarial rash, joint pain, recurrent fever, leucocytosis, elevated C-reactive protein (CRP), and monoclonal immunoglobulin (Ig)M or IgG gammopathy. In 2012, an expert conference elaborated the *Strasbourg criteria* for the diagnosis of Schnitzler’s syndrome and defined IgM or IgG gammopathy and urticarial rash as the two obligate criteria. Minor criteria are recurrent fever, objective signs of abnormal bone remodelling, elevated CRP level and/or leucocytosis, and a neutrophilic infiltrate in a skin biopsy (1).

We here report a case of Schnitzler-like syndrome without gammopathy in which we describe various mutations potentially related to inflammatory processes. A notable feature of this case is that we could demonstrate neutrophilic epitheliotropism in the skin biopsy (2), which additionally confirms the diagnosis of an autoinflammatory disease.

## Case description

**Figure 1:**
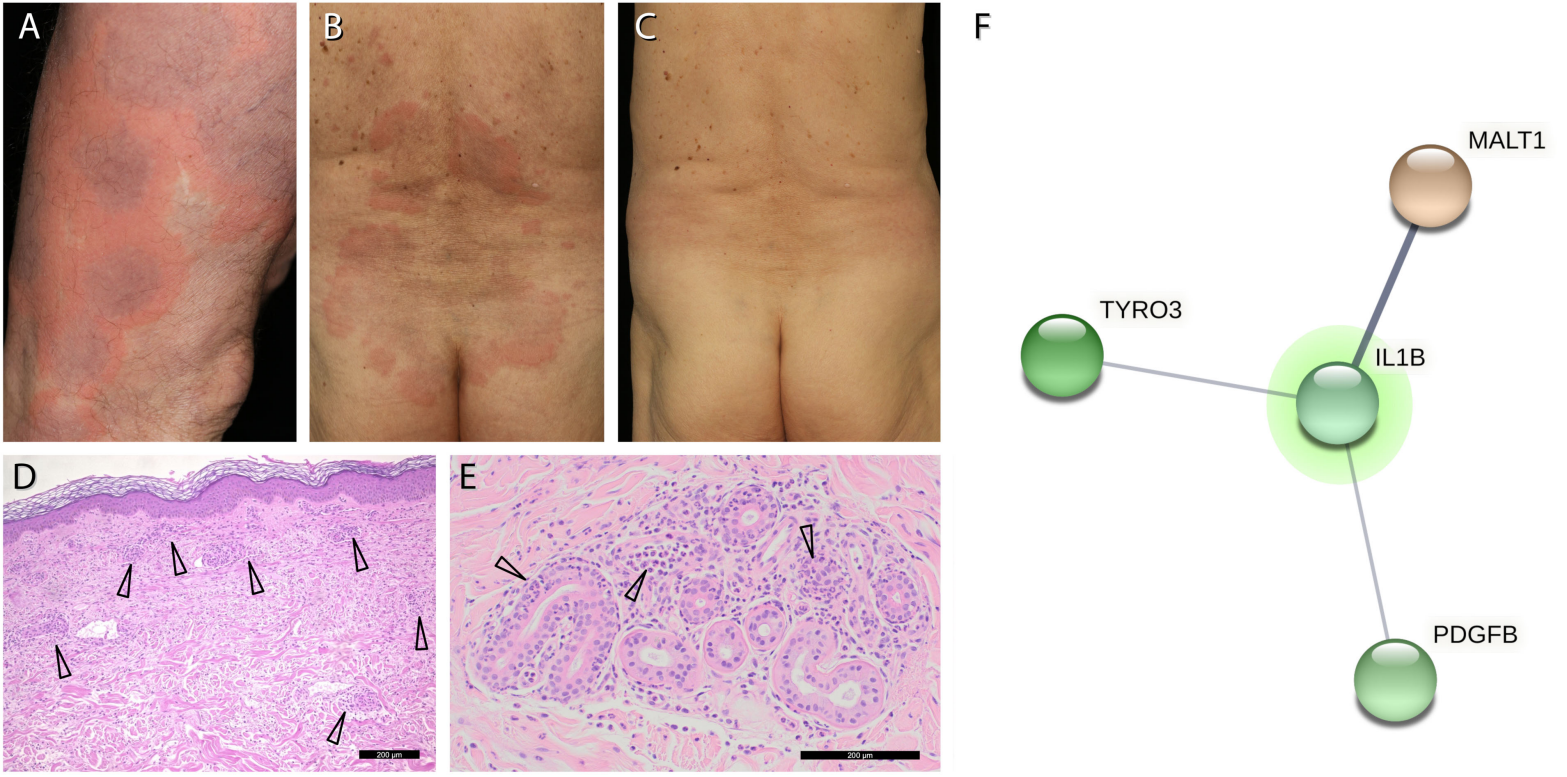
Clinical picture and genetic analysis. **(A, B)**, non-pruritic recurrent annular urticarial plaques on the extremities and on the trunk. **(C)**, resolution of urticarial plaques within hours in response to anakinra. **(D)**, Haematoxylin-eosin staining of a lesional skin biopsy shows a perivascular and interstitial neutrophilic infiltrate (arrows). **(E)**, Magnification of panel **(D)** with neutrophils in the epithelium and excretory ducts of sweat glands (arrows). **(F)**, interaction network of the 3 genes with putatively deleterious somatic mutations that may affect the function of IL-1β (*IL1B*) as primary mediator of inflammation in our patient: MALT1/ENST00000648670.1: c.167-2A>G (AF 13.6%, 17 of 120 reads), *TYRO3*/NG_033013.1: g.19447del (*AF* 18.5%, 28 of 151 reads) and *PDGFB*/NM_002608.4: p.Tyr30Ter (AF 13.9%, 11 of 79 reads). Putatively deleterious variations (*Dann* score >0.99 if available, *CADD phred* score >25, mutations detectable in at least 10 reads) were analysed for interactions using StringDB (URL: https://string-db.org, last accessed September 29^th^, 2022).

**Figure 2:**
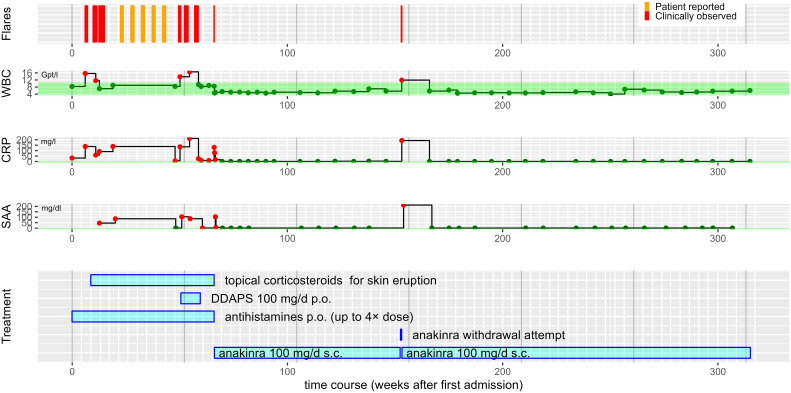
Overview of the patient’s disease course. Step plots of laboratory parameters over time aligned with bar chart of treatment modalities. With the exception of an immediate relapse during a single anakinra withdrawal attempt, the patient has remained free of inflammatory episodes for a follow period of about 250 weeks (~5 years) now. Different antihistamines including cetirizine 10 mg p.o were given up to 4x standard dose. Topical corticosteroids included methylprednisolone and clobetasol. WBC, white blood cell counts, in 1/nl. CRP, C-reactive protein concentration in mg/l. SAA, serum amyloid A concentration in mg/dl. The reference ranges are shaded in green. Red dots indicate values outside the reference range, dark green dots values within. The white vertical grid lines indicate months, the grey vertical grid lines years.

A 75-year-old man presented to our department with recurrent non-pruritic, not painful, annular urticarial plaques on the extremities and the trunk for 12 months (**Figure 1A**). The inflammatory episodes lasted for 7-10 days without severe general impairment, arthralgia or fever. Laboratory investigations revealed leucocytosis (up to 16.56/nl, ref 3.6 - 10.5/nl), neutrophilia (up to 13.05/nl, ref. 1.5 - 7.7/nl), elevated CRP (32-193 mg/l, ref. <5 mg/l) and serum amyloid A (SAA, 5-212 mg/dl, ref. <0.6 mg/dl) during the episodes of skin inflammation (**Figure 2**). Repeated immunofixation detected no gammopathy, and bone marrow biopsy did not reveal any pathologic changes. Histopathology of lesional skin showed a perivascular and interstitial neutrophilic infiltrate, initially described as consistent with the diagnosis of urticaria vasculitis (**Figure 1D**). However, neither high-dose antihistaminics (cetirizine 10 mg or desloratadine 5 mg up to 4× daily) over six months nor dapsone (100 mg/day) for three months effectively prevented the relapse of inflammatory episodes. Diagnostic workup for inflammatory foci, including gastro- and colonoscopy, transesophageal echocardiography, and full-body FDG-PET-CT, did not reveal any underlying infection, malignancy, or abnormal bone remodeling.

The strikingly schematic recurrence of the inflammatory episodes led us consider an autoinflammatory disease with features reminiscent of Schnitzler’s syndrome, albeit in the absence of IgM or IgG gammopathy. We thus initiated treatment with the interleukin (IL)-1 receptor antagonist anakinra (100 mg/day). Within five hours, the urticarial plaques resolved entirely (**Figures 1B, C**, **2**). The patient has remained in remission under continued treatment with anakinra 100 mg/day during follow-up for more than four years. Temporary discontinuation of anakinra for two days resulted in a relapse with re-emergence of cutaneous urticarial plaques accompanied by fever, joint pain, and elevated inflammatory markers (**Figure 2**).

Given the clinical presentation of our patient with Schnitzler`s syndrome features in the absence of gammopathy, we re-evaluated the skin biopsies. We found neutrophilic granulocytes located in the epithelium of sweat gland ducts (neutrophilic epitheliotropism), a distinctive feature of cutaneous manifestations found in autoinflammatory disorders (**Figure 1E**) (2). To unveil potential associated somatic and germline mutations, we performed whole exome sequencing (median coverage of exchange mutations 77×) from whole blood DNA at Novogene (Beijing, China). The patient agreed in accordance with the genetic diagnostics regulations and with the Declaration of Helsinki principles to the genetic analysis. Sequencing data in *fastq* format were mapped to the *hg19* human genome reference built using *bwa mem* (URL https://github.com/lh3/bwa, last accessed 13. March 2023) and further processed following *Genome Analysis ToolKit* (*GATK*) best practices for somatic variant calling (3–5). Variants were annotated using *DeepVariant* (URL: https://github.com/google/deepvariant, last accessed 13. March 2023) (6). No known pathogenic or high-scoring variation could be identified within genes associated with autoinflammatory diseases^1^, especially those listed in the INFEVERS data base (URL: https://infevers.umai-montpellier.fr/web/, last accessed 27. Feb. 2023) (7). To investigate whether any relevant gene variation found in the patient might influence IL-1β-driven inflammation, we used the online tool StringDB (8). This database combines connections between different proteins or genes found in different databases in a generic format. These connections include those found in databases on known interactions, co-expression, and genetic neighborhood, as well as connections found by text mining in Pubmed abstracts. We used multiple protein search as search strategy, with all 57^2^ high-scoring gene variations (CADD-Phred score > 25, DANN score (if available) > 0.99) and IL-1β as additional search term. The StringDB tool returned a network representing the (different) interactions between the proteins or genes we were interested in. This network revealed interactions of IL-1β with putatively pathogenic variations in MALT1 (MALT1 paracaspase), TYRO3 (TYRO3 protein tyrosine kinase) and PDGFB (platelet-derived growth factor subunit B) (**Figure 1F**), all of which are involved in cellular activation pathways and inflammation.

## Discussion

The first case of a Schnitzler-like syndrome without gammopathy was published in 2000 by Husak R et al. (9). In a current literature research, we found 8 cases in total with a similar constellation (**Table 1**). In nearly all cases, the criteria fever, increased CRP levels and/or leucocytosis were described. Neutrophil counts were not specified in most of the reports, although the *Strasbourg criteria* require determination of neutrophil counts greater than 10/nl. In our case, the patient did not report fever, except during the anakinra withdrawal attempt. In many of the earlier cases, bone changes were not properly objectified, as required. Only the subjective symptoms arthralgia and bone pain were described. In more recent cases, PET-CT was used to identify bone changes. In our case, we could not detect any abnormal bone remodelling, neither by PET-CT nor by determination of bone alkaline phosphatase. Concerning the minor criterion of neutrophilic infiltrate, the description of dermatohistopathologic findings do not always match a neutrophilic urticaria or neutrophilic urticarial dermatitis (NUD). Neutrophilic epitheliotropism has only been described in our case.

**Table 1:** Summary^1^ of published cases with Schnitzler-like syndrome without monoclonal gammopathy.

Age (years)	Sex	Chronic urticarial rash	Gammopathy	recurrent fever^2^ (°C)	Bone changes^3^	Neutrophilic infiltrate	Elevated WBC and/or CRP^4^	Treatment^5^	Outcome	Ref./Year
57	m	Severe recurrent urticaria, lesions lasting less than 24 h	IgM elevation to 6.5 g/l, no para-protein	“inter-mittent fever”	“arthralgia and bone pain”	perivascular infiltrates of lymphomononuclear cells and granulocytes throughout the dermis, accompanied by mild leucocytoclasis	CRP 133 mg/l; WBC 13.4/nl	AH, GCS, AZA, CLC, TRO, IFN, APAP, PPH	Not resolved	Husak (9), 2000
36	m	Non-pruritic urticarial lesions, lasting from a few hours to a few days, with residual pigmentation	IgM elevation to 3.46 g/l, no para-protein	≤40.0	“arthralgia and bone pain”	Neutrophilic urticaria	CRP n.d., WBC 19.8/nl	GCS, AH, NSAIDs, Thd, IFNα2b	Not resolved	Varella (10), 2005
58	f	Recurrent urticaria	Elevated IgG (20.1 g/l) and IgA (6.97 g/l)	≤38.5	“Arthralgias”	Edema of the superficial dermis and an interstitial perivascular mononuclear and polymorphonuclear cell infiltrate	CRP 61 mg/l WBC 9.3/nl	AH, GCS, NSAIDs, MTX, ANA	Resolved within 6 h under ANA	Treudler (11), 2007
58	f	Recurrent urticaria since the age of 9 years	Elevated IgA 703 mg/dl	No fever	“Joint pain”	Neutrophil infiltrate.	CRP 151 mg/l WBC 15.3/nl	MTX, SSZ, CsA, GCS, AH, ANA	Resolved within 3 h under ANA	Chu (12), 2010
62	m	Diffuse urticarial wheals, not painful, but extremely pruritis; lasting 2-3 days, with residual pigmentation	None (14 months)	“fever”	“Arthralgia”	Neutrophilic urticaria, no evidence of vasculitis	CRP n.d., WBC 21.1/nl	GCS, MTX, AZA, DDAPS, ADA, CLC, ANA	Resolved within 24 h under ANA	Urbanski (13), 2016
69	m	Urticarial rash, not painful, but with severe pruritus	None (3 months)	≤39.0	PET: bone-marrow hyper-metabolism	Neutrophilic and eosinophilic inflammation without evidence of vasculitis	CRP 136 mg/l;	ETA, AH, GCS, CLS, CsA, DDAPS, MTX, ANA	Resolved under ANA within 24 h; died due to pneumonia	Ahn (14), 2018
51	m	Recurrent urticaria	κ light chains elevated 47.2 mg/l; λ light chains elevated at 28.3 mg/l	“fever”	“joint and muscle pain”, sclerosis of pelvic bone, bone marrow osteosclerosis	Subepithelial edema and discrete interstitial and perivascular neutrophilic and eosinophilic granulocyte infiltration	CRP 46 mg/l; WBC 6.7/nl	OMA, ANA	Resolved under ANA within days	Henning (15), 2020
21	f	Non-pruritic urticaria with angioedema	IgM elevated to 2.57 g/l; no paraprotein	≤39.0	“arthralgia”, PET-CT: bone-marrow hyper-metabolism	Leukocytoclastic neutrophilic infiltration, minimal vasculitic aspects, minimal dermal edema	CRP 59 mg/l; WBC 22.9/nl	NSAIDs, GCS, CsA, OMA, ANA	Resolved under ANA within 24 h	Bixio (16), 2021
43	m	Recurrent urticarial rash	None	>39.0	Bone pain, leg pain, FDG-PET-CT: uptake of ^18^FDG in bone marrow of pelvis and femurs	Mild infiltration of lymphocytes, eosinophils, and neutrophils around blood vessels in the dermis.	CRP 61.8 mg/l; WBC 11.9/nl	GCS, AH, OMA, CsA, CAN	Resolved with 48 h under CAN	Fujita (17), 2021
75	m	Recurrent urticarial rash	None (72 months)	Initially: no fever	Initially: no bone pain and no arthralgia. No signs of enhanced ^18^FDG uptake	perivascular and interstitial neutrophilic infiltrate, neutrophilic epitheliotropism	CRP 193 mg/l; WBC 16,7/nl	AH, DDAPS, topical GCS, ANA	Resolved within 5 h under ANA	*this report, 2023*

^1^ modified and updated from Bixio R et al. (16) and Chu CQ (18) using selective literature search in PubMed, Google and Scopus. Cases with delayed development of gammopathy were excluded.

^2^ Strasbourg criteria require temperatures higher than 38°C. If temperatures were not documented, the symptom “fever” was put into quotation marks.

^3^ Strasbourg criteria require objective detection of bone changes by bone scintigraphy, MRI or elevation of bone alkaline phosphatase, with or without bone pain; description of subjective symptoms were put in quotation marks. n.d., not described.

^4^ Strasbourg criteria require neutrophils >10/nl and/or CRP >30 mg/l.

^5^ ADA, adalimumab; ANA, anakinra; AH, antihistamines; APAP, acetaminophen; AZA, azathioprine; CAN, canakinumab; CLC, colchicine; CsA, cyclosporine; DDAPS, dapsone; ETA, etanercept; GCS, glucocorticosteroids; IBU, ibuprofen; IFNα, interferon alpha (2b); IFX, infliximab; MTX, methotrexate; NSAIDs, non-steroidal anti-inflammatory drugs, not further specified; OMA, omalizumab; PPH, plasmapheresis; SSZ, sulfasalazine; Thd, thalidomide; TRO, trofosfamide.

Gammopathy may be lacking at the first manifestation of inflammation and skin symptoms, but develop during the course of the disease (19). In addition, (polyclonally) increased IgM levels were found in about half of the cases summarized by Bixio R et al. (16).

Schnitzler`s syndrome without IgG or IgM gammopathy described as Schnitzler-like syndrome in the literature is a potentially underdiagnosed entity (13–15). But the spectrum of Schnitzler-like syndromes may be not restricted to autoinflammatory symptoms with urticarial rash lacking gammopathy (20). The term MGARF (monoclonal gammopathy, arthralgia, and recurrent fever syndrome) describes a Schnitzler-like syndrome without skin involvement (20).

The fast response of Schnitzler-like syndromes lacking monoclonal gammopathy to anakinra was found in several other case reports (**Table 1**) (16). However, a fast response to IL-1β blocking drugs may not be specific for Schnitzler’s syndrome or Schnitzler-like syndromes (21, 22). In addition, IL-1 β blocking drugs may not always be effective for the treatment of autoinflammatory diseases with neutrophilic urticarial dermatitis, e.g., like VEXAS syndrome (23).

The *Strasbourg criteria* were developed to facilitate the diagnosis of Schnitzler syndrome. The disease is rare enough that it is helpful to use very specific and stringent diagnostic criteria. However, our case and previous case reports suggest that the criteria may be too stringent, and some cases of this disease may be missed. It took us about 1½ years before we decided to make the diagnosis of Schnitzler-like syndrome and initiate anakinra. During this period, we ruled out virtually all other diseases that could lead to an urticarial rash with a sharp increase in CRP and SAA levels. The prompt response of a disease flare after anakinra administration strongly confirmed our diagnosis. So did a recurrence of inflammatory symptoms during a withdrawal attempt from anakinra. Histologic specimens were re-evaluated during the follow-up, at which time we found the phenomenon of neutrophil epitheliotropism. This invalidated the previous diagnosis of urticarial vasculitis and also confirmed the diagnosis of autoinflammatory disease (2). During the follow-up period of almost 6 years, we did not detect the emergence of monoclonal gammopathy in this patient. The prompt response to anakinra — also suggested being a useful criterion for diagnosis by Gusdorf et al. (24) —and evidence of neutrophilic epitheliotropism support our diagnosis of Schnitzler-like syndrome without monoclonal gammopathy.

Neutrophil epitheliotropism is a pathognomonic feature of neutrophilic urticarial dermatitis (NUD). This allows distinguishing urticaria with neutrophil pattern from true NUD. A NUD with neutrophilic epitheliotropism is typical of autoinflammatory diseases such as Still’s disease, Cryopyrin-associated periodic syndrome (CAPS), and Schnitzler’s syndrome. However, neutrophilic epitheliotropism is also found in autoimmune diseases such as lupus erythematosus and primary Sjögren’s syndrome (2). Neutrophilic dermatoses are broadly divided into two classes: one form due to polyclonal hereditary activation of the innate immune system as in CAPS and Still’s disease, and a second form with monoclonal somatic activation of myeloid cells as in myelodysplastic syndrome or VEXAS syndrome. Urticarial lesions with neutrophilic infiltrate have been described in most cases of Schnitzler-like syndrome without gammopathy (**Table 1**). However, our case is the first in which neutrophil epitheliotropism is explicitly described in a Schnitzler-like syndrome, although this could have been overlooked in the previously described cases. So far, the pathogenic background of Schnitzler`s syndrome with its enigmatic interplay between monoclonal gammopathy and increased IL-1β secretion by monocytes and macrophages has remained elusive. Excess of IL-1β production is a hallmark of various autoinflammatory disorders with a monogenetic background and mutations of the NLRP3 inflammasome and other regulators of IL-1β processing. However, a genetic analysis failed to establish an association of Schnitzler`s syndrome with germline or somatic mutations in the *NLRP3* gene locus except in rare individual cases (15, 18, 25–28). Nevertheless, it is probable that Schnitzler’s syndrome is caused by an acquired mutation, like in VEXAS syndrome (29, 30), or in acquired Familial Mediterranean Fever (31) and acquired NLRC4-associated CAPS (32). Interestingly, a somatic NLRP3 mutation (NLRP3: c.1709A>G (p.Tyr570Cys)) identical to that reported in a Neonatal Onset Multisystem Inflammatory Disease (NOMID, or Chronic Infantile Neurological, Cutaneous and Articular Syndrome, CINCA) case was also found in a patient with a clinical picture closely resembling Schnitzler’s syndrome, and without gammopathy and without bone pain like in our patient (22). Therefore, it seems reasonable to search for somatic mutations in all patients with Schnitzler’s syndrome. In addition, the detection of somatic mutations requires sophisticated variant filtering algorithms. In our case, common variants (allele frequency above 0.001 in gnomAD or 1k genomes project) and variants with a variant allele frequency below 0.1 were removed. Additionally, variants with coverage less than 10x and with a CADD score below 20 were removed.

To search for causative mutations in our patient, we used a whole exome sequencing approach. However, we could not identify a pathogenic or putatively pathogenic mutation in genes currently known to be associated with autoinflammatory diseases. Based on the sequencing coverage, we estimate that we could detect pathogenic mutations with an allelic burden of about ≥5%. This may be simply insufficient. For comparison: in mastocytosis, an allelic burden of the cKIT:p.Asp816Val mutation of 0.01% can already cause symptomatic anaphylaxis (33). On the other hand, we could find variations in genes that interact with IL-1β that might have an impact on the inflammatory phenotype. One of these genes is PDGFB (platelet-derived growth factor B), which was found because it is co-mentioned along with IL-1β in PubMed abstracts. PDGFB is involved in cell proliferation, migration, wound healing and angiogenesis. It has been shown that PDGFB expression is associated with rheumatoid arthritis risk (34). The second gene is TYRO3 (Tyrosine-protein kinase receptor 3), which is mentioned along with IL-1β in PubMed abstracts and for which putative homologs have been found to interact in other organisms than humans. This receptor belongs to the family of TAM (TYRO3, AXL, and MERTK) family of receptors that are primarily expressed by immune cells. It plays an essential role in efferocytosis and the resolution of inflammation (35). The strongest interaction was found with MALT1, in terms of being mentioned in abstracts along with IL-1β, co-expression of putative homologs in other organisms and association in curated interaction databases. MALT1 (mucosa-associated lymphoid tissue lymphoma translocation protein 1) is the human paracaspase and involved into NF-κB activation. It has been identified as potential target for treatment of inflammatory diseases (36).

## Patient’s view

The patient himself reports that he is happy that we could find a solution for his disease which has remained stable for now about 5 years. He has no relevant side effect by anakinra, although he has noticed that his diabetes mellitus is noticeably better controlled since we initiated this medication. Anakinra is actually known to be effective in diabetes mellitus type II (37).

## Conclusion

In the cases reported so far, Schnitzler-like syndromes without gammopathy respond rapidly to treatment with IL- 1β inhibitors. Although the *Strasbourg criteria* are not met in such cases, these criteria provide guidance to consider the diagnosis of a late-onset autoinflammatory syndrome. Evidence of neutrophil epitheliotropism, an episodic course of the inflammatory symptoms, and a rapid response to probational administration of IL-1β inhibitors may help to confirm the diagnosis.

## Data availability statement

The raw data supporting the conclusions of this article will be made available by the authors, without undue reservation.

## Ethics statement

Ethical review and approval was not required for the study on human participants in accordance with the local legislation and institutional requirements. The patients/participants provided their written informed consent to participate in this study. Written informed consent was obtained from the individual(s) for the publication of any potentially identifiable images or data included in this article.

## Author contributions

ASW worked up the case, made photographs, composed figures and wrote the manuscript. AK, AF, HB and RJL performed bioinformatic analysis of sequencing results, discussed results and contributed to the manuscript. CR found the neutrophil epidermotropism and contributed the histopathological findings and interpretation. PL was involved into the diagnostic work-up of the patient and the decision about therapeutic strategies, and contributed to the manuscript. AR was involved into all stages of patient-care including diagnostic and therapeutic decisions, into work-up of sequencing samples and bioinformatic analysis, composed figures and wrote the manuscript. All authors contributed to the article and approved the submitted version.

## Footnotes

1 List of genes associated with autoinflammatory diseases used for searching pathogenic variants: ELANE, IL1RN, IL36RN, LPIN2, MEFV, MVK, NLRC4, NLRP12, NLRP3, NOD2, PSMB8, PSTPIP1, ADGRE2, NLRP1, WDR1, TMEM173, TNFRSF1A, ADA2, CECR1, TRNT1, TNFRSF11A, LACC1, PRF1, UNC13D, STX11, STXBP2, LYST, RAB27A, AP3B1, KIT, TNFAIP3, SLC29A3, SH3BP2, RBCK1, PSMB9, PSMB4, PSMA3, POMP, PLCG2, OTULIN, NLRP7, IL10, IL10RN, IL10RB, IL10RA, CARD14, APIS3, PTX1, ADAM17, ALPK1, AP1S3, CDC42, CEBPE, F12, IKBKG, NCSTN, PSMB10, PSMG2, RELA, RIPK1, SAMD9L, STING1, TRAP1, UBA1.

2 Complete list of high scoring genes in this patient: RFWD2, HEATR5B, SCRN3, TNIK, UBXN7, CNGA1, SLC9B1, KIAA1109, KIAA1324L, SPIN1, ARHGEF12, LIMA1, MDM1, OTOGL, MED13L, PABPC3, CKB, XAF1, FAM20A, MALT1, PDGFB, DIAPH2, STAG2, FMO6P, ATP6V1C2, FAM84A, FAM178B, CNOT11, TBC1D23, MOB1B, ZSWIM6, MARVELD2, RAPGEF6, CTC-432M15.3, SMAD5, RNF44, BCLAF1, BAG4, TMEM67, OPLAH, CRB2, IKZF5, GPR19, SCAF11, POLE, RXFP2, SSTR1, RMDN3, TYRO3, SPATA5L1, ZC3H18, SARM1, TMEM199, SLC46A1, CAPN12, ODF3B, SLC25A5.
